# Cultural identity central to Native American persistence in science

**DOI:** 10.1007/s11422-021-10071-7

**Published:** 2022-01-29

**Authors:** Nizhoni Chow-Garcia, Naomi Lee, Vanessa Svihla, Claira Sohn, Scott Willie, Maija Holsti, Angela Wandinger-Ness

**Affiliations:** 1 Department of Inclusive Excellence, California State University, Monterey Bay, CA 93955 USA; 2 Department of Chemistry and Biochemistry, Northern Arizona University, Flagstaff, AZ 86011 USA; 3 Organization, Information and Learning Sciences, University of New Mexico, Albuquerque, NM 87131 USA; 4 Department of Biology, Northern Arizona University, Flagstaff, AZ 86011 USA; 5 Department of Pathology, University of New Mexico, Albuquerque, NM 87131 USA; 6 Department of Pediatrics, University of Utah, Salt Lake City, UT 84112 USA

**Keywords:** Indigenous, Summer internship, Science identity, Cultural identity, Mentoring

## Abstract

Native Americans are the least represented population in science fields. In recent years, undergraduate and graduate level summer research programs that aimed to increase the number of Native Americans in science have made some progress. As new programs are designed, key characteristics that address science self-efficacy and science identity and provide supports for Native American students’ commitment to a scientific career should be considered. In this study, we used sequential mixed methods to investigate the potential of culturally tailored internship programs on Native American persistence in science. We analyzed surveys (*n* = 47) and interviews (*n* = 4) with Native American students to understand their perceptions of themselves in relation to science research and how summer research experiences might develop science identities. Based on regression modeling, science identity, but not science self-efficacy, predicted intent to persist in science. In turn, science self-efficacy and Native American identity predicted science identity, and this suggests cultural identity is central to Native American persistence in science. In interviews, students’ comments reinforced these findings and shed light on students’ reasoning about the kinds of science experiences they sought; specifically, they chose to participate in culturally tailored internships because these programs provided a sense of belonging to the scientific community that did not conflict with their cultural identities. Based on our analysis, we propose an Indigenous science internship model and recommend that agencies target funding for culturally tailored programs from high school through early-investigator levels as well as provide inclusive programmatic and mentoring guidelines.

Indigenous knowledge systems and more expressly, Indigenous science, is a living of right relations with lands, waters, and each other (Bang 2020). Western science traditions and the formal US educational system have spent the last 300 years stripping Native peoples of this worldview (Juneau 2001), imbuing power on a supposedly objective, culturally devoid, and human-dominant perspective (Deloria and Wildcat 2001). Such dissonant worldviews, as well as widespread distrust of formal US schooling, have impacted Native American higher education achievement (Shotton, Lowe, and Waterman 2013), so much so that Natives are the least represented population and the least likely to graduate from college, with the overwhelming majority of bachelor’s, master’s, and doctoral degrees in non-science, technology, engineering, & mathematics (STEM), service-oriented disciplines (NSF 2016).

Faced with a science culture steeped in meritocracy and characterized by white, masculine values, and behavioral norms, women and students of color have had more difficulty thriving in undergraduate science than white men (Carlone and Johnson 2007). Prior literature on women and students of color in STEM has clarified that gender and culture matter (McGee and Bentley 2017); however, only the most recent literature on Natives in STEM, and more often those with Indigenous authors (e.g., Page-Reeves, Marin, Moffett, DeerInWater, and Medin 2019), approach their research from an inherently affirming, Indigenous perspective, and one which explicitly explores the interconnected nature of science and Indigenous identity.

In working to increase the number of Native scientists, we approach this study with a critical Indigenous research lens. Critical Indigenous research (1) actively engages in a decolonization process; (2) emphasizes Indigenous knowledge and values; and (3) works toward self-determination and sovereignty (Claw et al. 2018). This lens directs analysis toward the ways in which systemic and structural forces shape Native American science educational attainment, as well as honors how Indigenous ways of thinking work to sustain and empower Native peoples, particularly as it relates to science (Kovach 2009).

## Positionality

Given that the first two study authors are Native American, our use of a critical Indigenous lens offers an epistemological interpretation that is more deeply personal and potentially insightful. The following is a brief summary of our stories—who we are, where we come from, and how we locate ourselves within our work; we have included this for the authors who participated in data collection and analysis only.

*Nizhoni Chow-Garcia*. I identify as an urban, mixed Native woman. On my maternal side, I am Diné and of the Tódích’íi’nii (Bitter Water People) and Totsohnii (Big Water People) clans. On my paternal side, I am Chinese. I grew up in California in a city east of Los Angeles and spent most summers visiting family on the Navajo Nation. Although I did not grow up on the reservation, I remain closely connected to my familial and cultural ties and have spent most of my professional years supporting Native students and communities as the Director of a Native student support program in higher education while at the same time engaging in research that actively works to increase the number of Natives in higher education and in STEM in particular.

*Naomi Lee*. I am from the Seneca Nation of Indians and of the bear clan. My siblings and I were raised on our original lands, the Seneca Cattaraugus reservation, located in western New York. Throughout my academic journey I was often the only Native American represented at my university and in my field of study (chemistry and biochemistry). Since completing my doctorate degree, I strive to change that pattern by engaging Native American students into STEM and research at an earlier stage in their academic journey. Thus, I am actively involved with various programs targeted toward Native American high school and undergraduate students. These include the NINDS (National Institute of Neurological Disorders and Stroke) of the National Institutes of Health (NIH), UPN (Undergraduate Pipeline Network) and CURE (Continuing Umbrella for Research Experience) at the University of New Mexico. More recently, I am the co-founder for the Summer Program for Yakama Students (SPYS) at Pacific Northwest University of Health Sciences and Heritage University in central Washington. In addition, I am the founder and co-director for the Cultural and Academic Research Experience (CARE) program at Northern Arizona University. Both SPYS and CARE are supported by NINDS with the goal of preparing high school students for college and research careers in STEM.

*Vanessa Svihla*. I identify as white and descended from early English and French colonizers/settlers and more recent German and Bohemian immigrants to the USA. I was raised in a highly diverse community, with 80 languages and dialects in my elementary school. I received a master’s degree in geology, but I became labeled as part of the “leaky pipeline” when I chose to pursue a PhD in the learning sciences. These experiences fostered in me a desire to be part of designing more just futures.

## Framework

**Fig. 1:**
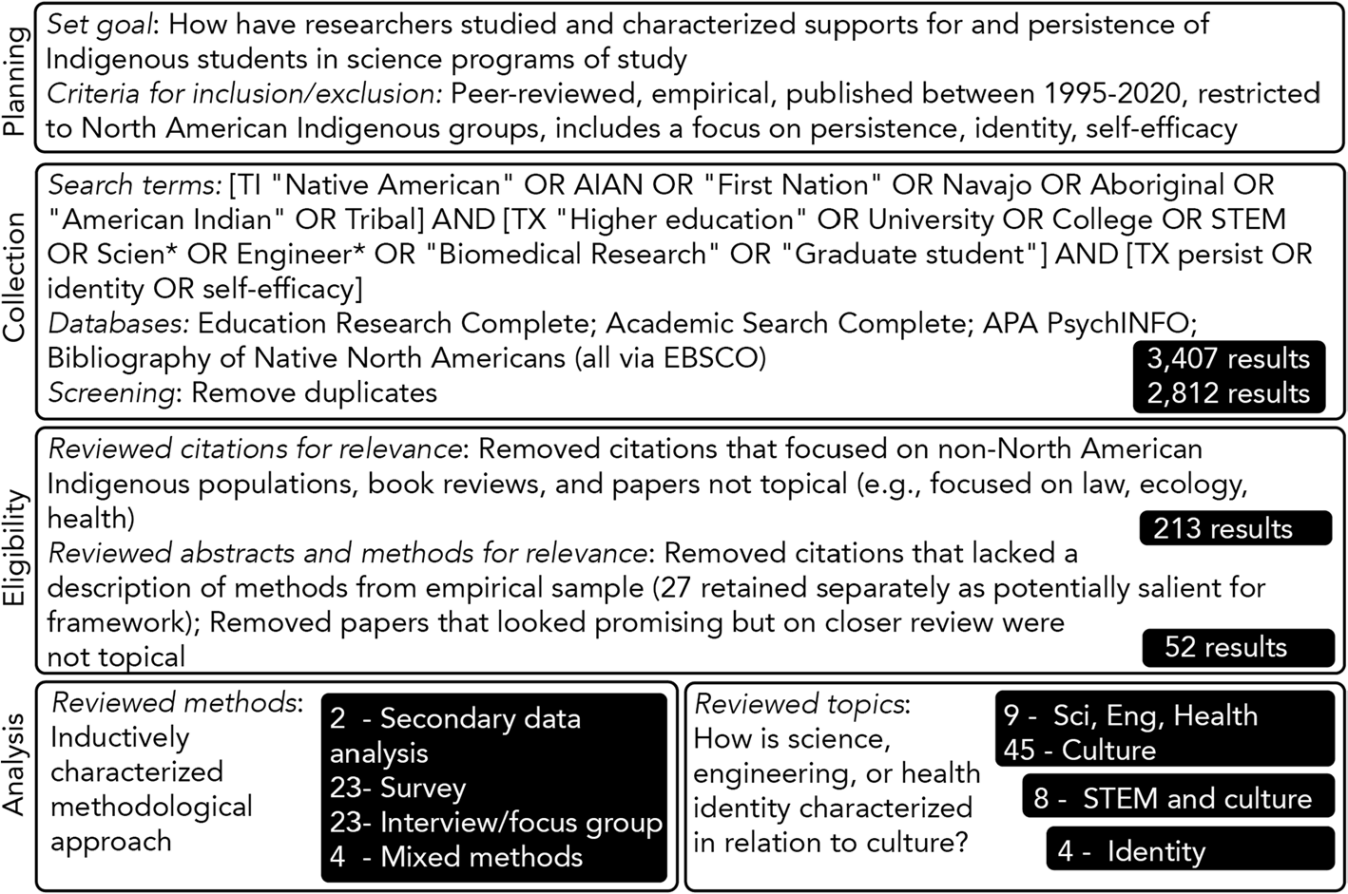
Overview of systematic literature review

The purpose of this study is to better understand and characterize, how and in what ways cultural and psychosocial processes increase Native American students’ commitment to science. We recognize STEM to be a construct largely utilized by governmental agencies and thereby subject to certain limitations (e.g., funding and specific disciplines); however, we interpret STEM to be a defined need in Indian Country, especially given the paucity of Natives with STEM degrees (NSF 2016) and the limited research exploring this population. We conducted a systematic literature review in order to situate our work within the results of studies that specifically focused on the development and persistence of Indigenous students in STEM and health fields (Fig. 1). We identified few studies that investigated science, engineering, or health identity formation among Indigenous students at the secondary or post-secondary levels, especially from a cultural lens. We thereby also draw on the rich literature on students of color in STEM to provide more context for psychosocial and cultural processes that influence STEM success. We use the term students of color—inclusive of Blacks, Native Americans, and Latinx, but not Asian Americans, who tend to have higher test scores, participation and persistence rates in STEM—rather than emphasizing deficit terminologies such as minority and underrepresented populations. We use the terms American Indian Alaska Native (AIAN) to align with summer research internship language, and thereby, governmental agency terminology. We more often prefer and engage the terms Native American, Native, Indigenous, and specific tribal status, recognizing that Native Americans may prefer to self-identify with their tribal status rather than broad pan-ethnic identities such as Native American (Horse 2001). To demonstrate the political nature of tribal sovereignty and identification, we intentionally capitalize the term Indigenous.

### Models of science persistence

We build on existing models of science persistence in higher education that have included students of color, finding that science identity and science self-efficacy directly predict intent to persist. We describe these models, then contextualize science self-efficacy and science identity. Three similar models influenced the field and the current study: Hanauer, Graham, and Hatfull found that in science courses, project ownership, self-efficacy, science identity, scientific community values, and networking explained significant variance in intent to persist in science (Hanauer, Graham, and Hatfull 2016). Their participants were overwhelmingly from groups well-represented in science, with only 6% reporting otherwise, and no students identifying as Native American. Estrada, Hernandez, and Schultz, in a longitudinal study of students of color (including an undisclosed number of Native Americans, who were binned with other non-Latinx and Black students), likewise found that in the short term, self-efficacy, science identity, and scientific community values predicted intent to persist, but in the longer term, only science identity was predictive of actual persistence (Estrada, Hernandez, and Schultz 2018). Chemers, Zurbriggen, Syed, Goza, and Bearman (2011) proposed that science supports—participating in research experiences, being mentored, and being involved in the science community—affect psychosocial processes like science self-efficacy and identity, which in turn lead to persistence in a science career. They surveyed members of the *Society for the Advancement of Chicanos and Native Americans in Science*, and through structural equation modeling, they identified three mediators—science self-efficacy, leadership and teamwork self-efficacy, and science identity—as particularly impactful. Their study included Native students, who comprised 38 of their 327 respondents. In a further study of the same setting, they emphasized that research experience contributes to science self-efficacy, which in turn contributes to science identity; however, they also reported that the “model explained less variance in identity as a scientist for participants who identified as African American, Latino, or Native American” (Robnett, Chemers, and Zurbriggen 2015).

Other studies have likewise suggested variability in the degree to which science identity and self-efficacy tend to predict persistence. For instance, in engineering, first-generation Latinas showed higher self-efficacy yet low sense of identity in engineering (Verdin and Godwin 2018), whereas for students from groups well-represented in engineering, self-efficacy and identity were significantly and positively correlated (Schar et al. 2017). Likewise, Byars-Winston et al. (2016) found that overall, science self-efficacy predicted science identity, but identified intersectional subgroups that showed significant differences, namely that Latinas had higher science identity than other groups. In a study focused on African-American HBCU college students, researchers found that science identity indirectly predicted science achievement and science self-efficacy mediated the relationships between cultural identity measures and science achievement (White, DeCuir-Gunby, and Kim 2019). Students’ of color science identity significantly predicted their grades in a science course, a relationship not found for other students in the course (Ballen, Wieman, Salehi, Searle, and Zamudio 2017).

We argue these varied results suggest a continued need to focus on culture in the preparation of science researchers in specific ways. First, cultural and disciplinary norms organize perceptions, often blinding those in privileged roles to the ways they benefit from their privilege (Collins and Bilge 2016). STEM cultures, including educational experiences, tend to mirror the oppression seen more broadly in society, in part driven by norms related to holding a positivist epistemology and the pursuit of objectivity (Aikenhead and Ogawa 2007). Together, these reproduce inequities in STEM education systems and professions. In contrast, connecting science to Indigenous students’ cultural experiences and epistemologies invites them to take a more expansive view of who produces STEM knowledge, allowing them to see themselves and their communities as already part of the production of STEM knowledge (Bang and Medin 2010). While qualitative studies have contributed to and deepened this latter view (Page-Reeves, Marin, DeerInWater, and Medin 2017), prominent models of science persistence reflect the influences of the former.

Specifically, the three models of science persistence in higher education raise questions about the role of science self-efficacy in forming science identity, key supports for forming both science self-efficacy and identity and suggest a need for research that investigates the experiences and development in culturally informed ways, and especially with attention to Native student persistence in higher education. Thus, research suggests complex and intersectional relationships exist between cultural identities and common predictors of science persistence, which we detail next.

### Science self-efficacy predicts persistence in science

Self-efficacy describes how confident an individual is in their ability to accomplish specific practices (Bandura 1977). Self-efficacy is typically linked to success (Pajares 1996) and often directly (Hanauer et al. 2016). For instance, Native Americans who have higher college self-efficacy are likelier to persist in college (Gloria and Kurpius 2001).

Science self-efficacy focuses on confidence related to specific science practices, such as collecting data, writing research questions, and using research literature (Chemers et al. 2011) and is typically measured through survey questions that ask individuals to assess their ability (“I can”) or their confidence in their ability (“I am confident that I can”) to perform a specific task. White men tend to report higher science self-efficacy, and this contributes to their higher rates of persistence (Leslie, McClure, and Oaxaca 1998) and success, including in attaining faculty positions (Landino and Owen 1988). In contrast, people of color hold less than 10% of science faculty positions (Fisher et al. 2019). Thus, self-efficacy directly influences persistence in science (Hurtado et al. 2007), making the study of factors that influence science self-efficacy important, particularly for Indigenous students.

### Science identity predicts persistence in science

Prior research clarified that science identity predicts students’ intent to become a scientist (Hanauer et al. 2016). Science identity is the degree to which students position themselves and feel positioned by others as a scientist (Hanauer et al. 2016). In this way, identity is double-sided work, dependent on an individual recognizing themselves as having membership in a group and on others recognizing them as belonging to that group (Holland, Lachicotte, Skinner, and Cain 1998). This notion of recognition has been cited as central to science identity, with evidence that a senior scientist who fails to recognize women of color as members can have a deleterious impact on their science identity formation (Carlone and Johnson 2007).

Identities are inherently relational and reinforce the concept of belonging. In Native cultures, relationships are the foundation to learning environments and are, moreover, the basis of tribal communities (Cajete 1994). This term—belonging—is reflective of an Indigenous worldview of our interrelatedness, interdependence, and mutual responsibility to all living beings (Deloria and Wildcat 2001). Science identity, thereby, must be understood to be more intimately connected to belonging and more integral to Native persistence in higher education (Flynn, Duncan, and Jorgensen 2012).

Support for understanding science identity in concert with Native identity comes from studies that have explored accounts of success primarily from the point of view of Native professionals. For instance, Dvorakova (2018), a non-Indigenous scholar, interviewed 40 Indigenous faculty (17 of whom worked in STEM fields) to understand how they navigated negative stereotypes. Participants explained that their cultures and worldviews empowered them to put effort into disproving stereotypes by sharing their own experiences or serving as a counter-example. Similarly, analysis (by a team that included Indigenous scholars) of interviews with 38 Indigenous medical students and physicians sought to identify facilitators and barriers in their pathways (Sanchez, Poll-Hunter, Stern, Garcia, and Brewster 2016). They found that the opportunity to serve as a role model to younger Native students was motivating, as they hoped such students would have better experiences en route to their careers. Jordan and colleagues (including two Indigenous scholars) interviewed 20 Navajo engineers to understand their work as engineers, finding that they centered Navajo behaviors like showing respect to Elders and valuing listening in their work (Jordan, Foster, Anderson, Betoney, and Pangan 2019). They reported this as both a tension and opportunity during their educational pathways, as they encountered cultural norms in engineering that seemed to conflict with as well as be compatible with their values.

Page-Reeves et al. (2017) and (2019) provide the most extensive qualitative research on Native professionals in STEM. Their research substantiates Native identity as key to success among Native STEM professionals and offers a more retrospective investigation of lifelong pathways, especially evident in their concept of “wayfinding,” which emphasizes that “through the experience of finding a path for themselves within the context of the dominant science education system, Native STEM professionals are creating new spaces *for being Native*” (Page-Reeves et al. 2019a, b, p. 185).

These studies, shared from the perspective of Native professionals, affirm that cultural identity and worldview can serve as a resource as Indigenous students find their ways toward success and build science identities. Such forged identities—that of one’s science and cultural identities—and the role this dynamic plays in Native STEM higher education success are at the heart of our project, and we aim to extend the work of Page-Reeves et al. (2019a, b, 2017) by focusing on Native students engaged in this wayfinding within their higher educational journeys with the hopes that we may have a more immediate impact on their higher educational experiences.

As we return to the discussion of science and cultural identities, we must consider how science identity has traditionally been studied as developed in school settings, and how dominant, hegemonic science instruction often presents science as an individual, brilliant, linear process—often with an outcome that is well understood prior to starting the investigation. This representation can create a sense of conflict or misfit if students’ first attempts at scientific investigation are not successful. For instance, Indigenous students who more strongly valued communal goals reported lower sense of belonging, which in turn corresponded to decreased intent to persist after just one semester of STEM coursework (Smith, Cech, Metz, Huntoon, and Moyer 2014). In contrast, early research experiences that provide opportunities to learn about science processes and practices as iterative and improvable can help students of color see and engage in science as a community, thus helping them to envision themselves as scientists (Villarejo, Barlow, Kogan, Veazey, and Sweeney 2008).

This suggests that establishing links between Native American identity and science identity may be particularly powerful for helping to support Native students’ development of a science identity, especially as we consider programs and practices in higher education settings. In this way, students can see connections between facets of their identity, rather than feeling that they must hide aspects of who they are in order to participate in science (Tracy and Trethewey 2005).

### Culturally tailored programs contribute to Native success in science

Science education should support students’ navigation of multiple epistemologies, inclusive of Native ways of knowing and being (Page-Reeves, Marin, et al. 2019). Others recommended that programs involving Native students use Indigenous pedagogical approaches (Kahn et al. 2021) and complementary cognitive apprenticeship learning strategies such as instructional scaffolding and experiential and problem-based learning (McMahon, Griese, and Kenyon 2019). To this effect, the need to develop culturally tailored programs in tribal (Augare et al. 2017) and mainstream schools from primary (Masta 2018) through post-secondary education is recognized among academic and government institutions (Estrada et al. 2016). As such, some summer research programs encourage Native students to embrace their scientific and cultural identities, rather than treating them as separable or their cultural identities as irrelevant (Holsti et al. 2015). Some programs accomplish this by letting mentors know when students might need to return to their communities for religious ceremonies, providing Native mentorship, or creating activities that build on Native cultural traditions (Lee, Nelson, and Svihla 2018). Likewise, using an Indigenous model can foster a sense of belonging and competence as biomedical researchers (McMahon et al. 2019). Similarly, involving a Native elder as a co-educator in a science course may increase Native students’ science identity (Alkholy, Gendron, McKenna, Dahms, and Ferreira 2017).

### Research purpose

The purpose of the present study was to investigate Native students’ intent to persist in science fields. We conjectured that, drawing on past research, while science identity and science self-efficacy may be important, cultural identity would also play a role in Native students’ intent to persist in science. By considering Native students’ science commitment through an Indigenous lens, we also aimed to contribute insight for those who develop culturally tailored internship or research programs. Specifically, we sought to investigate how and in what ways cultural and psychosocial processes increase Native students’ commitment to science, guided by the following four research questions:

1. What cultural and psychosocial factors explain variance in Native students' intent to pursue a career in the sciences?
2. What characterizes Native students' identities as scientists and how do they situate themselves as belonging in a community?
3. What cultural and psychosocial factors explain variance in Native students' science identity?
4. In what ways do Native students attribute research internships and other science experiences as contributing to their sense of belonging in a science community?

## Methods

To address the research questions, we used a sequential mixed methods design to first assess Native student perceptions broadly using a survey, and then to understand specific perspectives in more depth using interviews (Creswell and Clark 2007). We see mixed methods studies as particularly potent because of their capacity to describe trends related to more situated accounts of particular phenomena (Creswell and Clark 2007). We also note how few—only four—of the papers in our review employed mixed methods. These studies have provided insight into both trends and particulars. For instance, Kant, Burckhard, and Meyers (2018) used a post-survey to characterize the impacts of a culturally responsive program on Native high school students’ interest in STEM careers and a post-focus group to understand how particular aspects of the program supported the students. In the former, students reported increased interest and beliefs that fields like engineering and science are important to their communities. In the latter, students described how activities that supported them to see science in their cultural experiences gave them a sense of pride. The authors connected such activities to the outcomes of the survey. Rawana, Sieukaran, Nguyen, and Pitawanakwat (2015) used pre and post surveys with five and interviews with 12 Native participants to inform the design of an Indigenous peer mentoring program. Similarly, Pidgeon, Archibald, and Hawkey (2014) collected 60 survey responses and held six sharing circles to both understand how a peer mentoring program supported graduate students to feel a sense of belonging and accountability, and compare experiences across sites. Smith et al. (2014) conducted surveys to characterize that Native students at the beginning of their university programs and those who majored in STEM both valued communal goals, which contributed to their sense of not belonging in STEM programs that presented in these degrees as highly individualistic. Interviews with 33 students provided insight into this issue, as many of the students expressed a clear desire to use their degrees to help their communities, which in turn suggests ways STEM degree programs could foreground communal value of STEM careers.

These studies integrate insights in varied ways, reflecting the diversity of practices used in mixed methods to pragmatically shape understandings of *what* and *how* (Creswell and Clark 2007). Following this approach, we conducted mixed sequential analysis, allowing the results of qualitative analysis of interviews to shape the decisions we made in quantitative analysis. Specifically, when the results from our first regression model differed from trends reported in studies of persistence in science (Chemers et al. 2011), we turned to our qualitative analysis—bolstered by the results from qualitative studies reported in our review—to model science identity. Given the minimal research on science persistence among Native American students in higher education (Chow-Garcia 2016), interviews that are exploratory in nature can contextually and substantively enrich the quantitative results. Given the consistency of our interviewees’ accounts, we treated their accounts of their particular experiences as informative in conversation with interpretations of our survey data.

### Participants & Settings

Participants included students from four summer research internships that provide culturally tailored programs for Native American students: NARI (Native American Summer Research Internship) at the University of Utah, NINDS (National Institute of Neurological Disorders and Stroke) of the National Institutes of Health, UPN (Undergraduate Pipeline Network) and CURE (Continuing Umbrella for Research Experience) both at the University of New Mexico. Students were 16 years or older, high school through graduate/medical students, and all were US citizens or permanent residents. Combined, the summer internships had 162 students from various racial/ethnic groups, and with a majority female (71% of the total, 68% of Native American respondents). Of those students, 47 students self-identified as Native American (NARI = 19; NINDS = 19; UPN/CURE = 9). For our study purposes, we restrict our analysis to the Native American students. Programmatic information on eligibility, training, feasibility, and recruitment can be found in Table 1.

**Table 1:** Programmatic information for each of the four summer programs that include recruitment efforts, research training, and services offered for student feasibility

	NARI	UNM CURE #	UNM UPN#	NIH/NINDS
# AIAN students (%)	19 (100%)	7 (100%)	2 (7%)	19 (25%)
Eligibility	College junior or senior, registered at least part-time, US citizen of legal resident	16 + years of age, registered in high school at least part-time, local resident	18 + years of age, registered in high school or college at least part-time, US citizen or legal resident	* 16 + years of age, registered in high school or college at least part-time, US citizen or legal resident
Research training	10-wk biomedical training and clinical shadowing program; all interns start the last week of May	10-wk basic science, public health, and biomedical training programs; all interns start the second week in June	10-wk basic science, public health, and biomedical training program; all interns start the second week of June	8–12 wk biomedical training in one of ~ 48–55 different NINDS labs or affiliated NIH labs in other institutes; start and end dates are dependent on student/lab availability
Feasibility	All interns receive a stipend; travel support and registration to a summer conference; housing on campus	Hourly salary; housing was not provided	All interns receive a stipend; housing for non-resident students; housing and required course registration were deducted from summer stipend	** AIAN interns receive a stipend based on academic standing; health insurance; travel support to/from the program; travel and registration to a fall conference
Recruitment of AIAN students	Local and national Offices of Student Affairs and Offices of Diversity; AISES and SACNAS; brochures through social media; word of mouth	Visits to partnering tribal communities, high schools, and colleges; social media; word of mouth	Visits to partnering colleges; AISES and SACNAS national conferences; social media; word of mouth	Visits to partnering tribal communities and colleges; AISES and SACNAS national conferences; social media; word of mouth

^#^ CURE students consisted of 12 AIAN HS and undergraduate students. The 7 AIAN undergraduates also were part of the UPN

^*^Due to changes to NIH policies, as of 2018, all 16- and 17-year-old interns not from the area must be accompanied by a legal guardian

^**^As of 2018, all students not from the local area are now required to find their own housing. However, students interested in the Health Disparities in American Indian and Alaska Native communities have housing available through American University

The programs shared a commonality in recruitment, using word-of-mouth, social media, and representation at two Native American professional organizations—*American Indian Science and Engineering Society* (AISES) and the *Society for the Advancement of Chicanos and Native Americans in Science* (SACNAS). Each program provided culturally tailored experiences for Native American students with professional networking, social networking, and cultural inclusivity events (Table 2). Nearly all events sought to include Native American professionals and culture in many activities. Formal mentor pairing (Native American and non-Native American) methods varied between each program but all programs aimed to encourage peer-peer mentoring among the Native American students.

**Table 2:** Methods used by each program for mentor pairing, professional networking, social networking, and cultural inclusivity

	NARI	UNM CURE/UPN*	NIH/NINDS
Mentor pairing	Eight weeks prior to arrival, the participants were provided information about each of the research mentors and corresponding research project and ranked their top five. Program Administrator interviews each accepted applicant to ensure good placement	Program Administrators identified available mentors in the UNM School of Medicine, Cancer Center, and main campus. Lab mentors selected applicants based on their research interests, availability, and qualifications	Participants were encouraged to identify research mentors from the NIH websites. Program Administrators identified qualified NIH mentors and placed interns based on applicant’s interests
Professional networking	Laboratory-specific activities that vary across the campus; interactions with AIAN community and tribal leaders through the Utah Division of Indian Affairs	Weekly luncheons with AIAN professionals from UNM and outside organizations in the NM area	Laboratory-specific activities; visits to the CNAY; luncheons with senior AIAN professionals outside of NIH; local AISES chapter events; SACNAS potluck during NIH “Native Visit Week”; HHMI 3-day phage program at UMD
Social networking	Visits with the local Urban Indian Center of Salt Lake’s Summer Youth program	Weekend trips	Laboratory-specific activities; weekend trips to Smithsonian museums; BBQ with local AIAN professionals
Cultural inclusivity	Traditional blessing from the Confederated Tribes of Goshute’s spiritual elder; students and program administers participate in various national conferences and organizations throughout the year: SACNAS, AISES, American Diabetes Association: Scientific Sessions (ADA), Native Research Network (NRN), Association of American Indian Physician (AAIP), National Congress of American Indians, International Meeting on Indigenous Child Health, American Academy of Pediatrics	Calendar projects that includes the interns’ research in relation to their communities- calendars are provided to the students at the end of the summer; sponsorship to present their summer research at AISES or SACNAS in the fall	Journal club that includes literature related to AIAN health disparities; end-of-summer dinner with traditional foods prepared by the students; traditional blessings from the students in their Native Language

^*^AIAN students participated in the same activities as the CURE students; Other UPN activities were not provided by the program coordinator

For additional information, please contact the individual program coordinators via the contact information online

### Data collection

At the beginning of the summer internship, each program coordinator administered the electronic survey via an e-mail (Google Forms™). All survey responses were anonymous. The survey included subscales on science identity, science self-efficacy, and intent to persist in science, drawn from previous validation studies (Hanauer et al. 2016), which were themselves based on earlier measures (Estrada, Woodcock, Hernandez, and Schultz 2011). The cultural identity subscales were also modified from previous cultural incongruence items among Chicano/a/x students (Gloria and Kurpius 1996). We omitted subscales from the survey that focused on specific course-based science experiences because such questions would not be meaningful at the start of the internship. This included 10 questions on project ownership, 5 questions on emotions experience in a science course, and 5 questions about whether they shared their experience in their science course with others.” We also included questions related to cultural identity, drawn from the Lighting the Pathway study (Echohawk, Ondrechen, Megginson, Cornelius, and McClanahan 2014). Each question was Likert-scaled using a 5-point strongly agree to strongly disagree scale, with three to five items measuring each construct. Sample questions include:

- *Science self-efficacy*: I am confident that I can use technical science skills (use of tools, instruments and techniques)
- *Science identity*: I have a strong sense of belonging to the community of scientists
- *Cultural identity*: I believe that a career in my STEM field is compatible with my cultural values.
- *Intent to persist*: I intend to become a scientist.

The survey also included demographic questions (gender identity, educational attainment for self and parent/legal guardian, and race/ethnicity). Data on educational attainment were captured categorically (some high school education, high school graduate, current undergraduate or associate’s degree, bachelor’s degree, current graduate student degree). Participants could check all race and ethnicity groups that applied (white, non-Hispanic; Hispanic, Chicano/a/x, and Latino/a/x; Native American, American Indian, Alaska Native; Asian; Pacific Islander/Native Hawaiian; African or African-American; other). We included any respondent who identified as Native American, including those who also selected other race or ethnicity groups.

As an exploratory follow-up, and to gain further understanding, we chose to recruit Native American students from the same sample surveyed following completion of the internship. We deliberately sought to conduct interviews 1–3 months after the internship concluded such that students had time to reflect on the impact of the internship on their following activities (i.e., classes, research). One author (Lee) had a preexisting and ongoing relationship with many of the interns and with all of the internship directors. Kovach (2009), in describing the importance of and reciprocity of Indigenous relationships in research, emphasizes the relational aspect of sampling as directly connected to the trustworthiness of the researcher and that people choose to be a part of one’s research because they know the researcher and the researcher’s reputation. She, thereby, recruited students by word-of-mouth and social media in Fall 2017. While no students specifically declined, few volunteered. Four women volunteered to be interviewed—one who remained on campus following the internship and three who attended the AISES national conference held in Denver, CO. Of the interviewees, Chumani participated in one summer internship, Kara and Madalyn in four, and Johona in five (names are pseudonyms).

While we attempted to recruit a larger representation of students, including men, we were not successful due in part to the geographic distribution of students. Nevertheless, we were undeterred, as it is not unusual for studies of Native Americans pursuing STEM degrees to have small samples sizes, especially given that Native Americans are the least represented in STEM, earning just 0.7 percent (1,521) of STEM bachelor’s degrees in 2012 (NSF 2015). As part of our systematic literature review, we identified 27 empirical papers that included interviews or focus groups. The sample sizes and durations varied greatly, with five papers including two or fewer Indigenous participants, yet offering insight into possibilities, revealing relationships, or challenging assumptions. Similarly, studies with a small number of interviewees have shed light on ways culturally responsive or Indigenous approaches can support Native students: based on interviews with two Native and three Hispanic students, Evans (2004) detailed ways a caring curriculum can address challenges these students encounter, including feelings of isolation and coping with racism; using a combination of interviews and journaling, data from one Native faculty and student provided insight into some of the ways they used resistance in navigating their identities in a predominantly white institution (Jaime and Rios 2006).

Thus, despite the small sample size, we see these accounts as valuable and capable of shaping our understanding of particular experiences of phenomena. Given that the four women provided accounts that were similar to one another in many ways, and also correspondent with accounts reported for Indigenous participants in informal high school programs (Kant et al. 2018) and STEM professions (Page-Reeves et al. 2017), we see their accounts as shedding light on particular ways Indigenous students have built forged identities in their navigation of science educational experiences.

We specifically chose to conduct the interviews in-person, on the University of New Mexico campus in Albuquerque, NM or at the AISES national conference held in Denver, CO, which Lee and Svihla attended. Because Lee had prior prolonged experience with the interns but lacked experience conducting interviews, we decided that Lee would introduce the student to Svihla, who reviewed the interview purpose and consent, and conducted all interviews. Semi-structured interviews included questions about what made the internship feasible and attractive, what they perceived of as quality mentorship, their earliest science experiences, and other experiences that affected their journey as a scientist. Interviews ranged from 18 to 27 min, were audio-recorded and transcribed (Descript^®^). The authors reviewed and corrected the transcripts as needed. As discussed, given both the very small sample size and interview length, interview results are exploratory and should be interpreted as a preliminary examination of the lived experiences of individual Native STEM students. We recognize the diversity represented by the 574 federally recognized tribes, as well as the diversity within cultures cannot be captured in our small sample, yet see these young women’s accounts as valuable in providing particular points of view. We consider them in tandem with the results reported elsewhere (Page-Reeves et al. 2017).

### Statistical analysis

We calculated descriptive statistics for Likert survey items. We used ANOVA to compare survey responses by internship, anticipating that although the survey was completed at the beginning of the internship, applicants may have differed in systematic ways simply by how they selected their internship program. We found no evidence of clustering by internship (intent to persist, *F*(2, 59) = 1.66, *p* = 0.2; science identity, *F*(2, 58) = 1.66, *p* = 0.2); we therefore conducted ordinary least squares regression analysis to account for variance in (1) intent to pursue a career in science and (2) science identity. We confirmed that all assumptions for regression were met (Berry 1993) and report regression following APA norms. While several of the past studies that we build upon have used structural equation modeling and presented path diagrams to represent their results, our sample size, endemic to the nature of the topic of study, is not large enough to permit this type of analysis.

To find a parsimonious and comprehensive model, we tested solutions stepwise, retaining variables that explained significant variance; explanatory variables included demographics, science identity, Native American identity, and science self-efficacy. We additionally examined responses for significant differences, such as by gender and education level (e.g., high school, medical school), but found none.

### Qualitative analysis

We analyzed interview data using in vivo and values coding (Saldaña 2015). Each researcher independently analyzed two transcripts, initially focused on participants’ voice and values, and then identified dominant themes. We then met to compare themes across transcripts. While we refined the coding scheme somewhat, we found a high degree of alignment between coders and across participants. We also sought disconfirming evidence across transcripts. For instance, while interns mentioned recognizing failure as a learning opportunity, we reviewed their accounts of failure for counterexamples. We found the interns only shared examples that illustrated their understanding of failure as endemic to learning and the research process. The consistency across transcripts indicates that although we had a small sample, we reached saturation (Bowen 2008), perhaps because of the similarity of experiences these interns reported on in selecting and participating in internships. Yet, we also acknowledge that these four voices do not reflect the full diversity of experiences, especially as we consider the broader set of possible internships and educational opportunities available. To mitigate concerns about a small sample size, we also discuss our findings with those reported in our literature review.

The major themes included aspects of mentoring (i.e. good vs. bad mentoring, Native mentors, and mentoring of others), science identity, and Native American identity (Table 3).

**Table 3:** Themes from qualitative analysis

Theme (frequency)	Description
Sense of belonging (39)	Positions self as belonging to a group, such as saying "we" or indicates membership to a lab, school, tribe, etc
Good mentoring (37)	Describes an example of good mentoring
Support system (36)	Describes a support system, such as family and friends providing support to persist, or mentors or teachers providing specific supports
Native identity (34)	Mentions membership/affiliation with AIAN groups, communities or activities
Science identity (26)	Mentions membership/affiliation with science groups, communities, or activities
Home (21)	Uses phrase "the rez," references home community, or life/experiences on the reservation
Mentoring others (14)	Mentions providing mentorship to peers or others, in the past, present or future
Failure as learning (14)	Describes something learned as a result of failing to get an expected result, especially in lab experiments, but also in classroom-based activities
Giving back (14)	Explains wanting to give back to own group (family community, reservation, tribe, AIAN broadly)
Multiple internships (13)	Talks about doing more than one internship
Active/hands-on science (12)	Describes a hands-on, experimental set up, wet lab, activity in a lab or class, or describes everyday/informal science activities
Science is cool (10)	Mentions that science is cool or otherwise conveys enthusiasm for science
Native mentorship (10)	Mentions having had or desiring Native mentorship
Diversity matters (9)	Mentions the importance of diversity in science or comments to seeing diverse interns
Isolation (9)	Describes being isolated or being the only one, a minority among minorities
Poor mentoring (4)	Explains example of poor mentoring

## Results

After presenting the descriptive statistics, we report the results linked to each research question in sequence.

### Descriptive statistics

A total of 80 respondents represent an overall response rate of ~ 49% (80/162) across the four programs (Table 4). Of the 80 respondents, a majority identified as Native American (*n* = 47, 59%) with only 22% self-identifying as White or Asian and 8% as “other” that includes African-American, Hispanic/Latino, Native Hawaiian or other Pacific Islander. Of the total respondents, 71% identified as female (*n* = 56 of 80 total), with 68% of Native American respondents identifying as female (*n* = 32). Additional demographic information is provided for all respondents in Table 4, but remaining analyses focused only on students that self-reported as Native American only and Native American bi-/multi-racial.

**Table 4:** Demographic characteristics of all survey participants (*N* = 80, 49% of total possible) and AIAN participants (*n* = 47) from four summer internships

Characteristic	*N* total (% out of 80)	*N* AIAN (% out of 47)
Race/Ethnicity*		
AIAN	47 (59%)	47 (100%)
White or Asian	22 (28%)	–
Other	8 (10%)	–
Gender**		
Female	56 (71%)	32 (68%)
Male	23 (29%)	15 (32%)
*Program participation*		
NIH/NINDS	43 (54%)	19 (40%)
NARI	19 (24%)	19 (40%)
UPN/CURE	18 (23%)	9 (19%)
*Student’s academic status*		
High school graduate (or less)	17 (21%)	11 (23%)
Undergraduate (some college)	54 (68%)	30 (64%)
Bachelor's degree	7 (9%)	5 (11%)
Master's degree or higher	2 (2%)	1 (2%)
Number of prior summer internships**		
0	34 (43%)	20 (43%)
1	31 (39%)	18 (38%)
2	9 (11%)	5 (11%)
3 +	5 (6%)	3 (6%)
*Childhood community*		
Tribal lands or reservation*	28 (35%)	27 (57%)
Suburban	25 (31%)	6 (13%)
Urban	16 (20%)	10 (21%)
Rural	11 (14%)	4 (9%)
*Parent or legal guardian academic status*		
High school graduate (or less)	25 (31%)	20 (43%)
Associate's degree	11 (14%)	6 (13%)
Bachelor's degree	18 (23%)	11 (23%)
Master's degree or higher	26 (33%)	10 (21%)

*One student who responded "Other" for ethnicity self-reported living on tribal lands; two students chose not to respond

**One student chose not to respond

An equivalent percentage of Native American respondents were in the NIH/NINDS summer internship (40%) and NARI (40%), with only 19% in the UNM/UPN programs. Most of the Native American students were current undergraduates (64%) and 23% either just completed high school or were current high school students. Only 13% either recently completed their undergraduate degrees or were in a graduate program. Almost half of the students (43%) reported summer 2017 as their first internship with 38% indicating it was their second. The remainder (17%) of students reported two or more internships. Over half of the Native American students reported living on tribal lands or a reservation (57%) and approximately one-third in urban or suburban settings (34%). Approximately one-half of the students reported their parent or legal guardian’s academic status as a high school graduate or less (43%). However, 21% of the students reported that one or both of their parents/legal guardians had a Master’s degree or higher.

What cultural and psychosocial factors explain variance in Native American students' intent to pursue a career in the sciences?

We modeled variance in Native American students' intent to pursue a career in the sciences (on a scale of 1–5, *M* = 3.80; SD = 1.15) as a linear combination of summed scores tied to science identity (on a scale of 6–30, *M* = 24.17; SD = 3.98). In model 1, science identity explained significant variance in intent to pursue a career in the sciences, *F*(1, 44) = 57.37, *p* < 0.001. This model explained a significant amount of variance, *r*^2^ = 0.56, *p* < 0.001 (Table 5).

**Table 5:** Model of intent to pursue a career in science

	Unstandardized coefficients		Standardized coefficients	*t*
	*B*	Std. Error	*β*	
*Model 1: Intent to pursue a career in the sciences modeled as a function of science identity*				
Intercept	− 1.44	.70		− 2.05*
Science identity	.22	.03	.75	7.57**
*Model 2: Intent to pursue a career in the sciences modeled as a function of science identity and AIAN Identity*				
Intercept	−.93	.80		− 1.61
Science identity	.23	.03	.79	7.68**
AIAN Identity	− 06	.05	−.13	− 1.30
*Model 3: Intent to pursue a career in the sciences modeled as a function of science identity and science self-efficacy*				
Intercept	− 1.12	.86		− 1.30
Science identity	.23	.04	.80	6.25**
Science self-efficacy	−.02	.04	−.08	−.64

*p < .05; ** *p* < .01

In model 2, we added a variable for Native American identity (on a scale of 3–15, *M* = 12.50; SD = 2.45). Model 2 did not explain significantly more variance in intent to pursue a career in the sciences than model 1, *F*(2, 43) = 29.98, *p* < 0.001, *r*^2^ = 0.58, *p* > 0.05. We therefore omitted this variable from the model.

In model 3, we added a variable for science self-efficacy (on a scale of 7–35, *M* = 28.54; SD = 3.98). Model 3 did not explain significantly more variance in intent to pursue a career in the sciences than model 1, *F*(2, 43) = 28.50, *p* < 0.001, *r*^2^ = 0.55, *p* > 0.05. We therefore omitted this variable from the model. These models suggest that variance in science identity accounted for approximately 56% of variance in Native American students’ intent to persist in science, but that Native American identity and science self-efficacy did not directly explain variance in intent to persist in science.

These models suggest that students who possess stronger science identities are likelier to report that they intend to pursue a career in science, and this aligns with findings in studies using similar methods (e.g., Chemers et al. 2011). However, our models also suggested that variance in science self-efficacy did not predict intent to pursue a science career, a finding that does not align with the majority of studies (e.g., Chemers et al. 2011), many of which included students from well-represented groups (Hanauer et al. 2016). Given that in studies of students of color (primarily Black and Latinx students) only science identity predicted longer-term persistence (Estrada, Hernandez, et al. 2018), and that research reporting on successful STEM professional’s accounts of wayfinding and forging intertwined science and Indigenous identities (Page-Reeves et al. 2017), we were curious to more deeply investigate Native American students’ identities as scientists using qualitative analysis of interview transcripts.

What characterizes Native American students’ identities as scientists and how do they situate themselves as belonging in a community?

Overall, and correspondent to prior qualitative findings (Page-Reeves et al. 2017), students’ identities as scientists were inextricably linked to their identities as members of Native communities and tribes. When describing their identities as scientists, they commonly referenced their Native identity and sense of belonging. They likewise discussed belonging as it related to their home communities, their research labs, and to the broader scientific communities, often in interconnected ways.

*Native identity and belonging.* All four students recognized being Native as part of their identity. This came out in their introductions, as they introduced themselves by sharing their tribal affiliation(s). The students all made references to the Native experience, such as using the phrase “on the rez.” Even Kara, who explained that she had not grown up on a reservation, talked about connections to her culture and a sense of isolation at being away: “This was my first time not being primarily surrounded by Natives and being so far away from home, and I felt like the minority of minorities… I still felt slightly isolated and I felt like my mentors didn’t understand necessarily, my Native identity there.”

*Native science identity and belonging.* All four students discussed their science identities as being motivated by their Native identities. For instance, Kara argued, “We need more Natives treating Natives… I got to help my people… and that’s how I ended up at the Native American Research Internship.” Johona explained that her community “is known for like, like, um with facts or like we have a lot of high teen suicide rates, and um so that’s pretty much like why I wanted to go to public health.” Madalyn explained, “I want to be a pediatrician. And also doing research on diabetes and childhood obesity that is something near and dear to my heart because of my rez… that’s the highest cause of death… my grandma struggles with it and my grandpa struggles with it. And so that’s my goal—to give back and also because growing up on the rez I never had a consistent pediatrician… so I want to be able to go back eventually… and build trust with children especially in the Native community…” In these comments, we see these young women position themselves as future science professionals who serve their Indigenous communities, and this finding concurs with the results of interview studies with successful professionals who described this as a key motivator (Page-Reeves, Cortez, et al. 2019).

Students situated themselves as belonging to their lab or the broader science field, yet erstwhile connected this to their Native identities and familial connections. For instance, Kara explained, “a lot of what makes me feel like a scientist is being in the lab specifically … and then being validated by my family, like, my family, like ‘wow, you’re in the lab, and you’re curing cancer. You’re so cool. You’re a scientist,’ and so that kind of, that’s a huge feeling like a scientist thing.”

Madalyn, when reflecting on the increased numbers of Native American students in her program over three years, explained that “going from seeing only five other Native Americans to seeing me and thirty other Native Americans and Latinas” is like “seeing this whole new world beyond the reservation lines. I thought that was pretty cool.”

Native American students viewed science as an important part of who they are and made clear that it is not in conflict with their cultural integrity. Thus, students’ identities as scientists are interconnected to their Native identities and sense of belonging in science. We note that these interpretations are correspondent with findings reported elsewhere. Specifically, Page-Reeves et al. (2017) and (2019) showed that successful Indigenous professionals forged intertwined identities, and in particular, noted that while there was great diversity across the particular accounts of their wayfinding, all of these successful professionals strongly expressed a sense of their Indigenous identity (Page-Reeves et al. 2017). Indeed, their accounts of wayfinding en route to their successful careers (Page-Reeves, Marin, et al. 2019a, b) are visible in our interviewees’ in-progress accounts. This suggests that our interviewees, while they faced their own particular journeys, were already on the path these successful professionals described and were engaged in forging their intertwined Native and science identities. Based on this qualitative analysis, paired with past research showing that self-efficacy is typically tied to science identity, we decided to conduct further regression modeling to explain variance in Native American students’ science identity.

What cultural and psychosocial factors explain variance in Native American students' science identity?

Variance in students’ science identity (on a scale of 6–30, *M* = 24.17; SD = 3.98) was modeled as a linear combination of summed scores tied to science self-efficacy (on a scale of 7 to 35, *M* = 28.54; SD = 3.98). Overall, students reported high science self-efficacy, but those with prior internship experience had significantly higher self-efficacy, *t*(60) = 2.04, *p* < 0.05. In model 1, science self-efficacy explained significant variance in science identity, *F*(1, 44) = 28.76, *p* < 0.001. This model explained a significant amount of variance, *r*^2^ = 0.38, *p* < 0.001 (Table 6).

**Table 6:** Model of science identity

	Unstandardized coefficients		Standardized coefficients	*t*
	*B*	Std. Error	*β*	
*Model 1: Science identity modeled as a function of science self-efficacy*				
Intercept	6.21	3.38		1.84
Science self-efficacy	.63	.12	.63	5.36**
*Model 2: Science identity modeled as a function of science self-efficacy and AIAN Identity*				
Intercept	1.34	3.85		0.35
Science self-efficacy	.62	.11	.63	5.49**
AIAN identity	.42	.18	.29	2.32*

*p < .05; ** *p* < .01

In model 2, Native American identity (on a scale of 3–15, *M* = 12.50; SD = 2.45) was added. Model 2 explained significantly more variance in science identity than model 1, *F*(2, 43) = 18.51, *p* < 0.001, *r*^2^ = 0.44, *p* < 0.05.

Thus, Native American students who expressed a strong sense of science identity also had higher science self-efficacy and a stronger sense of Native American identity. This model aligns with findings from qualitative analysis that suggested that science identity is intertwined with Native American identity. This less direct route to persistence situates Native identity as a resource for developing science identity, in line with research that accounts for such work as a form of wayfinding (Page-Reeves, Marin, et al. 2019a, b).

In what ways do Native American students attribute internship and other science experiences as contributing to their sense of belonging in a science community?

In interviews, Native students elaborated on the significant role mentors played in contributing to their sense of belonging in science. Native American students noted that they benefited from mentors who were approachable and ready to support and value their contributions. This support came in various forms, described in similar ways by all students interviewed. Specifically, they described supportive mentors as those who provided active hands-on experiences; those who emphasized relationship building and belonging; and those who valued diversity and Native American identity. For those students who had a Native American mentor, specifically, they mentioned how impactful the experience was. Each of these is detailed below.

*Good mentors engage Native American students in active, hands-on science.* All students mentioned the importance of active, hands-on science experiences. Madalyn had multiple research experiences and evaluated her experiences and mentors according to whether or not she participated in an active, hands-on manner. She recounted that in her first summer research experience, she was “mostly doing data sheets and working on Excel” and shared that her mentor “found ways to make it seem like I didn’t know what I was doing and kind of like belittled my intelligence… [which] discouraged me a little from maybe applying the next year because I didn’t want to be treated like that. But then the next year I got put into a lab working with HTLV-1 and my mentor was completely incredible.” Likewise, Kara’s tone elicited affirmation and excitement when describing her active, hands-on science research experiences, such as when she “worked under one of my organic chemistry professors where I helped develop a protocol for extracting ethanol from corn stock.” As Chumani spoke about working in the lab and coming to understand the work she was doing, she explained, “now I know I absolutely want to be here, and I know that I absolutely belong here.” While much research has argued for the value of such experiences in supporting learning, we see alignment here with research on Native STEM professionals’ identity work being connected to having personal agency (Page-Reeves, Marin, et al. 2019a, b).

*See potential and develop it.* Mentors can support Native American students simply by seeing potential and building on that potential. Even seemingly small moves left an impression on Johona, who explained “I had like one specific like chemistry professor that was like ‘Hey. I really think you should do this. Here’s what it’s like.’ [He] laid it all out for me, explained the program and it’s like, […] those few like teachers in high school that like show you, like ‘You can do this.’”.

Madalyn described how a mentor helped her take up an agentive role in science by seeing failure as part of the process of science, which in turn made it safer to take risks and fail: “So this may not come off as, like, supportive, but my first summer at the University of South Dakota my mentor, he wasn’t really like a ‘I’m going show you everything.’ He’s like, ‘I want you to figure it out to test yourself.’ And me being who I am was like, ‘No, like this makes me anxious, like I don’t want to mess up’ and he was like, ‘That’s what science is… you’re going to mess up. I did not get here in my career because everything went smoothly.’ I feel like that was the best advice anybody could have given me.” As with offering opportunities for hands-on science, when mentors see and develop potential, students have chances to exercise their personal agency, which others have argued is a critical component of becoming an Indigenous scientist (Page-Reeves, Marin, et al. 2019a, b).

*Relationship building and belonging.* Students affirmed the power of relationship building and belonging fostered by mentors. Kara shared: “I was supported by other grad students that I met there and the mentors and directors of the program really wanted me to succeed and they- we still keep in contact, so they still really care and stuff like that…” In discussing her application and search for graduate schools, Kara declared that “wherever I end up there needs to be a support group for Natives… and [when] interviewing at Utah I was like okay, I’m already family and like I’m meeting up with them during my interview weekend. I get to see them and they’re like, ‘You’re doing amazing, you’re going to do great in grad school wherever you go’ and it’s like okay, I’m going to come here now because you say this to me all the time; it’s going to make it that much easier.” Kara explained “something that has made me feel like things are working and are making me happy are things like having a sense of community with the other students so making sure that there are planned activities.”

When talking about how she got her first internship, Madalyn described that “I got the internship through my chemistry teacher, and I excelled at that and I got published… and he thought, you know, I had the potential to do so and I feel like I proved him right…” This same mentor met with her outside the classroom, took her to conferences, and connected her to new people and internship programs. He was the “one who really believed in me” As others have noted, social relations are central to wayfinding (Page-Reeves, Marin, et al. 2019a, b). Mentors who not only see and develop potential, but do so from a relational stance can reinforce Native students’ sense of belonging even as they navigate their own paths.

*Supportive of diversity and Native identity.* Students recognized the importance of mentor support for diversity in general and their Native identity in particular. Kara emphasized that “My more recent mentors at Utah have really been very supportive. So my PI is a white old man but he understands like that being Native is my identity and he supports my endeavors in that. So you know I’m at AISES so he lets me come to these conferences. We were just awarded a grant that supports diversity in the lab, so like now I’m free to him. So that’s really exciting. So he’s always been really supportive of it. He hasn’t played a role in any sense of how or what my Native identity is at all, but he supports the idea.”

Mentors enhanced the students’ experience by connecting the research to students’ identity and/or community. As Johona talked about her mentor, she explained that early on, she confessed to her mentor, “I do’'t get this, like, my school’s not this advanced” to which her mentor replied, “Okay. That’s fine.” She related a later conversation with the mentor, which occurred as they reflected on working with depressed patients in their research. She had noticed a patient say “I live in three worlds” and thought “I say I live in two worlds” as a Native person. She brought this up, and her mentor was appreciative of this, acknowledging that she had not even noticed it, and that she was grateful for Johona’s perspective.

*Native mentorship.* For the students who experienced Native mentorship, this shared identity had a profound and lasting impact. Madalyn disclosed, “My mentor was completely incredible like also because she was Native American and so she could like understand my experiences, and we could like talk about those things together and like I was more comfortable talking to her and she’s incredibly intelligent so anything and everything that she asked of me, she explained in a way I can understand it.”

Similarly, Chumani shared an influential conversation she had with a Native mentor who talked to her about imposter syndrome or feeling like one does not belong: “You made it, but you still feel like you don't belong. And that's exactly where I was. ((laughs)) Sometimes I still feel like that, but just uh with, with her being in the lab being in this really diverse lab and telling me like she goes, ‘I don't feel that way.’ She tells me that uh she says 'I belong here and I worked my butt off to get here. And I know what I'm doing.’ … She still ((up tone)) exists. She exists. She does the job- um, really makes me feel like- like I can keep pushing the outsider syndrome away.”

## Discussion

Past research has commonly viewed Native American people as a statistically insignificant group, citing numbers too small to be studied (Shotton et al. 2013). This has often led to Native American people being lumped together with other students of color, made especially apparent when one turns to the science literature, despite noted variance by racial/ethnic subgroups (Byars-Winston et al. 2016). Alternatively, and most notably, this study highlights Native American culture by providing a lens into how culturally tailored internship programs can contribute to Native American students’ sense of science identity, self-efficacy, and intent to persist in science fields. Specifically, we investigated how and in what ways cultural and psychosocial processes increase Native American students’ commitment to science.

Study findings. Quantitative analyses show that Native American students’ science commitment was best predicted by science identity, which in turn was predicted by science self-efficacy and Native American identity. Thus, Native American students who expressed a strong sense of science identity also had higher science self-efficacy and a stronger sense of their Indigenous and cultural identity. This model aligns to findings from qualitative analyses—both our own and correspondent with others’ research as noted previously—that further support that science identity is intertwined with Native American identity. More specifically, student’s identities as scientists were inextricably linked to their identities as members of Native communities and tribes, and when, in interviews, describing their identities as scientists, the four women commonly referenced their Native identity and sense of belonging. They likewise discussed belonging as it related to their home communities, their research labs, and to the broader scientific communities, often in interconnected ways. In commenting on supports, not only did they acknowledge research mentors who fostered their sense of belonging in science, they also cited support from their Indigenous communities.

Our findings align with the results of several qualitative studies on Indigenous success in science. First, prior research on successful STEM professionals highlighted that while they navigated diverse and particular experiences, Indigenous identity served as a resource, and through wayfinding (Page-Reeves, Marin, et al. 2019a, b), became forged with their science identity (Page-Reeves et al. 2017). Our results extend this prior body of work by illuminating that students at comparatively early stages of their professional lives have already begun this journey of wayfinding and forging. Our quantitative results also extend these findings, suggesting that, as noted by many qualitative studies, cultural identities are salient in understanding science persistence.

These findings therefore suggest that influential models of science persistence that served as a foundation to the current study may be enhanced by incorporating cultural identity. For instance, Hanauer et al. (2016) focused on project ownership, self-efficacy, science identity, scientific community values, and networking in science courses with a majority of students from groups well-represented in science. When extending this model to populations that include Native students, including measures of cultural identity may provide greater clarity about the impact of specific course-based interventions on intent to persist. Likewise, Estrada et al. (2011) found that science identity—more so than other factors—predicted persistence. Incorporating measures of cultural identity, in our study, elucidated a role for self-efficacy, though more research is needed to understand how these factors operate longitudinally. Finally, adding a focus on cultural identity to Chemers’ et al. (2011) model could provide greater capacity to explain students’ of color persistence. Our mixed methods approach bridges the common psychosocial models of persistence with Indigenous views of this work as a form of wayfinding by treating Native identity as a resource.

Others have proposed that culture and caring environments—much like interactions students in our study described—matter when considering persistence of diverse scholars in science (Estrada, Eroy-Reveles, and Matsui 2018a, b). Likewise, high school performance and test scores did not predict persistence for Native American students (Benjamin, Chambers, and Reiterman 2010), while the “ability to adopt new traits while maintaining a traditional perspective may be a characteristic of persisters” (p. 37). This may be because Native American students tend to hold stronger cultural connections to their communities than their non-Native American peers (Okagaki, Helling and Bingham 2009) and to view their families as a top factor in completing college (Guillory and Wolverton 2008). For Native American students, strong cultural identity serves as an emotional and cultural anchor that promotes self-confidence and even a sense of purpose, where “they know who they are and why they are engaged in mainstream education” (Huffman 2010, p. 171). Thus, programs that support students to connect their Indigenous identities to what they are learning provide better support (Jordan et al. 2019), and this has been found in studies of Native American student academic success in college (Huffman, Sill, and Brokenleg 1986). Researchers have issued calls for better articulation of ways that higher education and Native American communities can provide culturally tailored supports, such as encouraging students to draw on traditional spiritual resources as sources of strength (Jackson, Smith, and Hill 2003). Doing so may prevent some of the strife successful Native scientists have described, including needing to work to overcome negative stereotypes and experiences of distancing or dissociating themselves from their cultural identities (Dvorakova 2018).

**Fig. 2:**
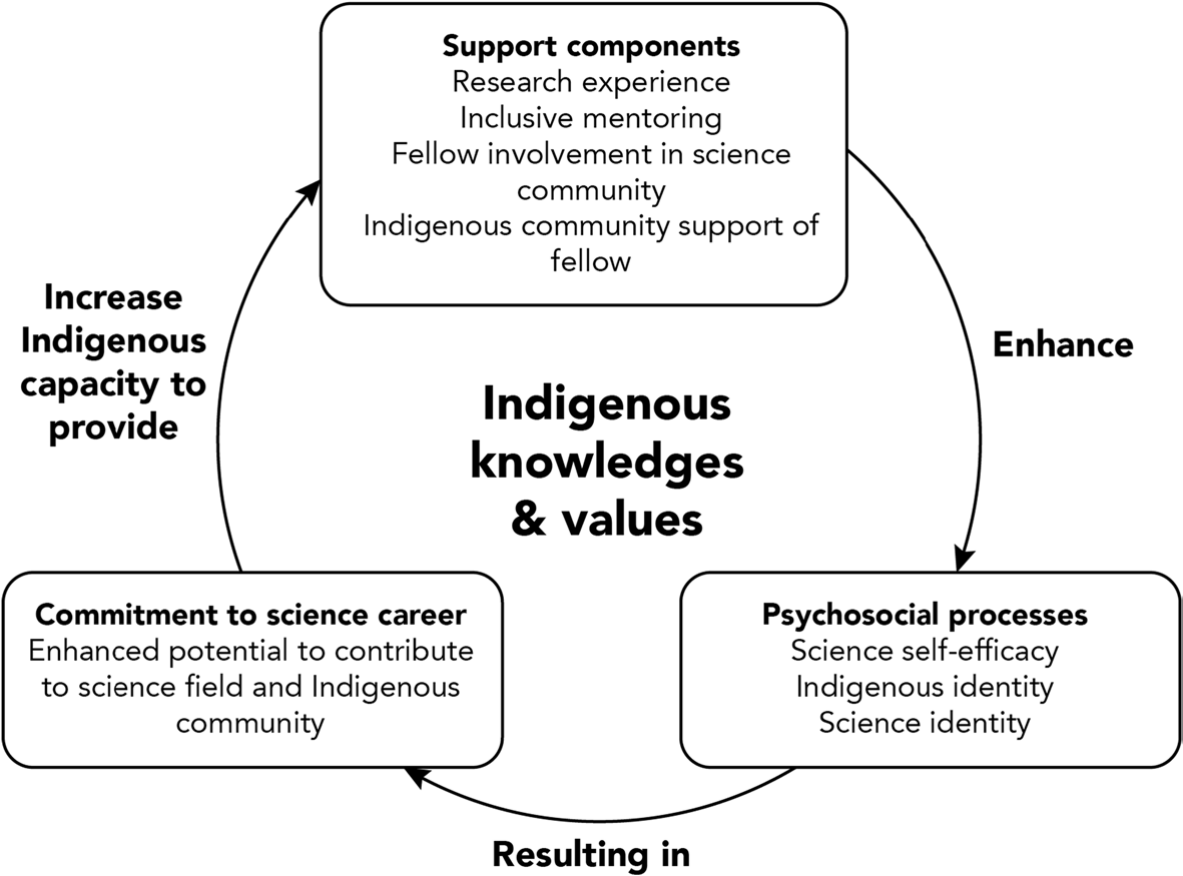
Proposed Indigenous science internship model (ISIM) to increase Natives in science

Use of a critical Indigenous lens. As reflected in the quantitative analysis and reinforced by the qualitative analysis, the linkage of Native American cultural and science identities is essential to Native American science commitment. Given such findings and the study population as a whole, it is necessary to examine such conclusions with a critical Indigenous research lens. While qualitative studies have previously provided evidence that Native American cultural and science identities are intertwined (Page-Reeves et al. 2017), this knowledge has not been broadly taken up in approaches that use quantitative methods. Westernized models, such as Chemers and colleagues’ (2011), are linear and seldom integrate culture in a meaningful manner. As such, it is necessary to move beyond Westernized models to one that centers Indigenous knowledges and values by acknowledging an interconnected, relational epistemology, which the authors propose as a guiding framework, the Indigenous science internship model (ISIM) (Fig. 2). At the core of this model are Indigenous knowledges and values. With this centering, an Indigenous lens must be integrated into understanding how support components, psychosocial processes, and commitment to science careers create an interdependent, yet self-determining system that reinforces and enhances capacity.

When informed by Indigenous pedagogy, a research experience should utilize a “learn by doing” model (McKinley, Brayboy, and Castagno 2008). This is evident in one of the major qualitative themes—the incorporation of “active, hands on science” and even in the notion of “failure as learning,” which implies an ongoing experiential component that is not only tolerant of errors, but situates these as learning opportunities. Because Indigenous science comprises the processes of knowledge acquisition through human experience in the natural world (Cajete 2000), transmitted from one generation to the next, especially orally, through daily social and cultural interactions (Ogwawa 1995), mentoring and building that relationship is integral for Native American students. Consistent with the broader literature, McMahon’s (2019) findings indicate that for Native American undergraduate research students, “mentors played a critical role in cultivating relational attachment and significance, competency, achievement, and self-determination within a communal learning environment” (p. 102). This provides insight into why student interviews (and corresponding themes) emphasized the importance of relationship building by research mentors, and the impact a Native mentor played in their experience.

Chemers and colleagues (2011) use the term community involvement to reflect opportunities outside the classroom or lab, such as social events and conferences, that might be available for students to develop a sense of being part of the scientific community. To support Native American students, the concept of community involvement must be more expansive so as to include and engage the broader community (family and tribal nations) and the students’ wish to “give back to their tribal community” and be supported by it in the process. For example, in 2015, the NINDS students attended former First Lady Michelle Obama’s speech sponsored by the Center for Native American Youth (CNAY) and the Aspen Institute (see Table 2). More recently, the NINDS program invited families and tribal leaders to Bethesda, MD, to participate in the end-of-summer research symposium. The tribal leaders expressed the empowerment it brought to their community by witnessing their Native American students speak of their research alongside all the other non-Native American students. The NARI students visited the Urban Indian Center in Salt Lake City and frequently met with tribal leaders. The UPN/CURE students participated in feast days hosted by the local Pueblo communities and created educational materials on cancer research projects conducted by each student and shared them with their tribal community members. In addition, each program included cultural, peer- and near-mentors, as well as opportunities to serve as a mentor to others in their own communities. While these are just a few examples, each program ensured frequent community engagement and professional networking via luncheons and panels with Native American professionals.

As noted in the findings, the incorporation of these program support components enhances Native American psychosocial processes of science self-efficacy, Indigenous identity, and science identity. As discussed above, Indigenous pedagogical aspects of learning are inherently experiential, and when Native American students engage in this type of “learn by doing” science guided by a supportive mentor, their science self-efficacy increases. Further, when program support components center Indigenous identity such as through engagement with tribal communities (e.g. visiting the Urban Indian Center in Salt Lake City), having mentors that support Indigenous identities, and even engaging Native mentors, Native American students’ sense of belonging increases—further reinforcing their identities as scientists.

Centering Indigenous identity in the program support components enhances psychosocial processes, that in turn result in an increased commitment to a science career. Qualitative findings, especially considered with the findings of other studies, articulate a greater communal connection, namely one that is not only deeply personal, but moreover relational. All four of the interviewees expressed this commitment. Kara stated that “We need more Natives treating Natives… I got to help my people… and that’s how I ended up at the Native American Research Internship.” Madalyn wants to be a pediatrician because she wants “to give back and also because growing up on the rez I never had a consistent pediatrician.” Reiterated in the qualitative data, Native American students’ commitment to a science career is not an isolated, independent endeavor; rather, it is a personal commitment to their Indigenous communities.

The use of critical Indigenous research provides a framework for understanding the importance of centering Indigenous knowledges and values in internships in order to increase Native Americans in science careers. This relational, interconnected way of being is not only decolonizing in its centering of Indigenous identity, but it is self-determining in its future and cyclical capacities to build and sustain Indigenous individuals and communities.

### Implications

In order to reach the goals presented by the Indigenous science internship model (ISIM), recommendations for supportive policies and practices follow.

Policy. As depicted in ISIM, in order to increase the number and capacity of Native Americans in science, scientific organizations such as the NSF and NIH can play a key role. First, such agencies can target funding for culturally tailored programs along the trajectory from high school to early-investigator levels. The summer programs assessed in the present study are but a few of NIH’s extramural programs offered for undergraduates. With the newly formed NIH Tribal Health Research Office (THRO), there may be many more opportunities for Native American student training across the NIH intramural organization such as the NINDS Health Disparities in Tribal Communities Summer Internship Program (SIP) (NINDS 2019). Second, providing programmatic and mentoring guidelines (Table 7), perhaps with supplemental funding to support effective implementation could buoy efforts to mentor Native American students (Werner-Washburne 2018). Our guidelines are based on the qualitative analysis that revealed aspects and behaviors of mentors that cultivate a greater sense of belonging through the linkage of cultural and science identities. Such guidelines would support non-Native American mentors to commit to effective Native American mentoring.

**Table 7:** Guidelines for supportive mentors of AIAN mentees

Guidelines	Practical examples
Engage mentees in active, hands-on science	Lead with the purpose or goal, rather than background reading Build mentee laboratory skills quickly to ensure participation in laboratory activities
Identify mentee potential and develop it	Show interest in mentee’s reasons for pursuing STEM Share the purpose and implications of the research so that the mentee can decide if they find it interesting Ask mentees for their own thoughts and interpretations
Develop relationships and sense of belonging with mentees	Show interest and care about their development Share examples of your own failures and uncertainties about your career path, and model how to learn from failed attempts
Support diversity in general and Indigenous identity in particular	Encourage mentee participation in their own cultural norms and traditions, even if it means time away from the lab Encourage mentees to share—if they feel comfortable doing so—the sources of their interest in STEM, as these are often intertwined with Indigenous identity Many Indigenous mentees wish to return and serve their communities. Be supportive of their choices
Recognize the significance of Indigenous mentorship and strive to provide this support for mentees	Encourage mentees to interact with other AIAN STEM professionals, both locally and at AIAN-focused conferences Talk to mentees about their futures as mentors

Practice. One way to apply policy efforts into institutional practice is through the use of the Indigenous science internship model (ISIM) that combines concepts from Chemers (2011) and cultural or social supports (Estrada, Zhi, Nwankwo, and Gershon 2019) (Fig. 2). Many ISIM components are not discipline or population exclusive and can be extended to programs supporting students in science and the health fields more broadly. Of particular value-added praxis to policy-makers, researchers, and any practitioners supporting Native American science students, is the ISIM support component on inclusive mentoring. Typically, internship mentors lack formal training on how to be a mentor, and further, may hold limited understanding of cultural differences that are salient when mentoring across cultures (Prunuske, Wilson, Walls, and Clarke 2013).

Regarding guideline 4, practical examples of how this might be accomplished include those previously mentioned regarding community engagement. Other examples include greetings and prayers in traditional language, art/dance to express themselves in ways common to their cultural upbringing, and talking circles (Flyswithhawks 1996). An additional aspect of mentors, and one that is intrinsically linked to the benefits of shared identity, is the significance of Native mentors. Although they are few and far between, they exist; and should study objectives be met, such future Native mentors may have the greatest influence yet.

### Limitations

Although based on the surveys used by Chemers (2011), Hanauer (2016) and Estrada (2019), the survey and modeling in this study necessarily differed, and this may have impacted the results. First, the addition of questions on Native American identity may have changed how students responded. Further research should be undertaken to validate the survey with additional respondents.

Second, because this study had a smaller sample size, ordinary least squares regression modeling was employed rather than path analysis. This means results are correlative in nature. Future research could expand on the findings of this study by exploring the role of cultural identity with a broader population and disaggregating results by ethnicity. Finally, the data analyses were pooled sample sets rather than within-program analyses. We specifically chose these analyses to avoid any program biases and individual-level data linkage to permit anonymous survey responses. Future research could also employ a pre/post design to track and compare the impacts of different programs. Such studies would provide better direction in terms of the kinds of cultural tailoring that are most beneficial.

While we were able to triangulate findings across and within data sources, additional interviewees may provide alternate perspectives, though additional interviews do not always guarantee this (Bowen 2008). Still, consideration of the full dataset suggests additional interviews could reveal more. Of particular note, because 68% of our Native American survey respondents are women and all the interviewees are women, whose experiences cannot be assumed to be the same as those of men (Crenshaw 1989), future studies should seek to engage more male participants in order to gain broader insight into their perspectives. Although we mitigated this issue somewhat by considering our qualitative results in tandem with others’ results, the small number of interviewees situates our work as exploratory in nature, yet provides a means to bridge more commonplace qualitative with quantitative studies. Future studies, especially those with external funding, may explore other ways to recruit more participants without concerns of coercion.

## Conclusion

Native American students pursuing science degrees are faced with unique challenges that often result in them leaving science fields, in part because they lack a sense of belonging in these fields or feel as if they have to choose between their Native culture or their science identity. By surveying and interviewing Native American students from four culturally tailored summer programs, this research sought to identify cultural and psychosocial processes that increase Native American students’ commitment to science.

For Native American students, commitment to science is fostered by science identity, which in turn is fostered by both science self-efficacy and Native American identity. Thus, cultural identity influences students’ sense of belongingness and persistence in science. Accordingly, science internships should be culturally tailored and provide mentors who both engage Native American students in hands-on aspects of research and value the perspectives Native American students bring (Table 7). Based on these conclusions, the Indigenous science internship model (ISIM) is proposed (Fig. 2). At the core of this model are Indigenous knowledge and values. With this centering, an Indigenous lens must be integrated into understanding how support components, psychosocial processes, and commitment to science careers create an interdependent, yet self-determining system that reinforces and enhances capacity.

We hope this research helps current and future programs better support Native American students, building on the understanding that Native American persistence in science fields ultimately depends on a science identity that is interwoven with one’s Native American identity. Ensuring that Native American students feel secure in expressing both their Native and science identities can build their sense of belonging within the science community; this in turn can increase the number of Native American students who persist in science and ultimately, increase their capacity to serve and sustain their Native communities. May we all, in our perspectives and practices, more deeply affirm the value of Indigenous science—the living of right relations with lands, waters, and each other (Bang 2020)—for it may hold the futurity of us all.
